# Donor-Derived Brain Tumor Following Neural Stem Cell Transplantation in an Ataxia Telangiectasia Patient

**DOI:** 10.1371/journal.pmed.1000029

**Published:** 2009-02-17

**Authors:** Ninette Amariglio, Abraham Hirshberg, Bernd W Scheithauer, Yoram Cohen, Ron Loewenthal, Luba Trakhtenbrot, Nurit Paz, Maya Koren-Michowitz, Dalia Waldman, Leonor Leider-Trejo, Amos Toren, Shlomi Constantini, Gideon Rechavi

**Affiliations:** 1 Cancer Research Center, Sheba Medical Center and Sackler School of Medicine, Tel Aviv University, Tel-Aviv, Israel; 2 Institute of Hematology, Sheba Medical Center, Tel Hashomer, Israel; 3 Department of Oral Pathology, School of Dental Medicine, Tel Aviv University, Tel-Aviv, Israel; 4 Department of Laboratory Medicine and Pathology, Mayo Clinic, Rochester, Minnesota, United States of America; 5 Tissue Typing Laboratory, Sheba Medical Center and Sackler School of Medicine, Tel Aviv University, Tel-Aviv, Israel; 6 Department of Pediatric Hemato-Oncology, Sheba Medical Center and Sackler School of Medicine, Tel Aviv University, Tel-Aviv, Israel; 7 Institute of Pathology, Tel-Aviv Medical Center, Tel-Aviv, Israel; 8 Pediatric Neurosurgery, Dana Children's Hospital, Tel-Aviv Medical Center, and Sackler School of Medicine, Tel Aviv University, Tel-Aviv, Israel; Hôpital Necker-Enfants Malades, France

## Abstract

**Background:**

Neural stem cells are currently being investigated as potential therapies for neurodegenerative diseases, stroke, and trauma. However, concerns have been raised over the safety of this experimental therapeutic approach, including, for example, whether there is the potential for tumors to develop from transplanted stem cells.

**Methods and Findings:**

A boy with ataxia telangiectasia (AT) was treated with intracerebellar and intrathecal injection of human fetal neural stem cells. Four years after the first treatment he was diagnosed with a multifocal brain tumor. The biopsied tumor was diagnosed as a glioneuronal neoplasm. We compared the tumor cells and the patient's peripheral blood cells by fluorescent in situ hybridization using X and Y chromosome probes, by PCR for the amelogenin gene X- and Y-specific alleles, by MassArray for the ATM patient specific mutation and for several SNPs, by PCR for polymorphic microsatellites, and by human leukocyte antigen (HLA) typing. Molecular and cytogenetic studies showed that the tumor was of nonhost origin suggesting it was derived from the transplanted neural stem cells. Microsatellite and HLA analysis demonstrated that the tumor is derived from at least two donors.

**Conclusions:**

This is the first report of a human brain tumor complicating neural stem cell therapy. The findings here suggest that neuronal stem/progenitor cells may be involved in gliomagenesis and provide the first example of a donor-derived brain tumor. Further work is urgently needed to assess the safety of these therapies.

## Abstract 2

Gideon Rechavi and colleagues describe the case of a boy with ataxia telangiectasia who developed a brain tumor after neural stem cell therapy.

## Editors' Summary

**Background.:**

Most of the cells in the human body are highly specialized (“differentiated”). The brain and the spinal cord, for example, contain two main cell types—neurons, which transmit electrical signals to and from the brain, and glial cells, which support and protect the neurons. If these essential neural cells become damaged or diseased, the body cannot replace them. Scientists think, however, that it might be possible to use “neural stem cell” transplants to replace the neural cells that are lost in neurodegenerative diseases (for example, Parkinson's disease) or damaged by strokes or trauma. Stem cells are undifferentiated cells that replicate indefinitely and that have the potential to develop into many different specialized cells. Pluripotent stem cells (which are able to develop into any kind of specialized cell) can be isolated from early human embryos; “multipotent” stem cells (which develop into only a few cell types) can be isolated from many differentiated tissues, including the brain. Human fetuses (unborn offspring from the end of the 8th week after conception) are thought to be a particularly good source of neural stem cells because many new neural cells are made in fetal brains.

**Why Was This Study Done?:**

Although stem cell transplantation might provide treatments for many debilitating diseases, some concerns have been raised over its safety. In particular, some experts fear that tumors might sometimes develop from transplanted stem cells. Tumor cells actually behave very much like stem cells—they divide indefinitely and they tend to be undifferentiated. It is very important, therefore, that every patient who receives a human stem cell transplant is carefully followed up to see whether any tumors develop as a result. In this study, the researchers describe a case in which multiple, slow-growing, donor-derived brain tumors formed in a patient after the transplantation of human fetal neural stem cells.

**What Did the Researchers Do and Find?:**

Beginning in 2001, fetal neural stem cells were injected several times into the brain and the fluid surrounding it of a boy with ataxia telangiectasia at a Moscow hospital. Ataxia telangiectasia, a rare disorder characterized by degeneration of the brain region that controls movement and speech, occurs when both copies of the *ATM* gene (human cells contain two copies of most genes) contain a genetic change that stops the production of functional ATM protein. In 2005, the boy had a magnetic resonance imaging scan at the Sheba Medical Center (Israel) because of recurrent headaches. The scan revealed abnormal growths in his brain and spinal cord. In September 2006, when the boy was 14, the spinal cord growth was surgically removed. This growth has never reappeared but the mass in the boy's brain has continued to grow slowly. The material removed from the boy's spinal cord contained both neurons and glial cells, the researchers report, and resembled a glioneuronal tumor. In addition, it contained both XX (female) and XY (male) cells and the tumor cells had two normal copies of the *ATM* gene. Finally, a technique called HLA typing showed that the tumor contained cells from at least two donors.

**What Do These Findings Mean?:**

These findings indicate that the growth in the patient's spinal cord was donor-cell derived and contained cells from two or more donors, at least one of whom was female. Although the growth in the patient's brain has not been examined, the multiple masses seen in this patient probably arose independently from transplanted cells injected at different sites, suggest the researchers. Importantly, the slow growth of the tumors and the well-differentiated appearance of the cells removed from the patient suggest that the tumors are relatively benign. Donor-derived cells might have been able to establish tumors in this particular patient because people with ataxia telangiectasia often have an impaired immune system and the immune system normally helps to reject tumor cells. Nevertheless, this first example of a donor-derived brain tumor developing after fetal neural cell transplantation is worrying and suggests that further work should be done to assess the safety of this therapy.

**Additional Information.:**

Please access these Web sites via the online version of this summary at http://dx.doi.org/10.1371/journal.pmed.1000029.

- The US National Institutes of Health provides information about stem cells and their potential uses
- The International Society for Stem Cell Research also provides information about all aspects of stem cells for the public and for professionals
- The US National Human Neural Stem Cell Resource provides specific information about neural stem cells for the public and for scientists
- The US National Cancer Institutes has a fact sheet about ataxia telangiectasia

## Introduction

Research in recent years has demonstrated that neurogenesis constitutively occurs in specific regions of the adult mammalian brain and that there are substantial numbers of multipotent stem/precursor cells from many parts of the brain that may be used therapeutically [1–4]. The fetal brain, characterized by active neurogenesis, has been suggested to be a promising source of therapeutic neural stem cells [5]. Neural stem cells are being investigated as potential therapies for neurodegenerative diseases, stroke, and trauma [1–4,6–9]. Such cells have also been suggested as potential therapies for infants and children affected by genetic and acquired diseases characterized by neurological deterioration [1,5].

Neural tissue-derived stem cells, phenotype-specified progenitor cells derived from pluripotential embryonic stem cells (ESC), and neural cells derived from various transdifferentiated non-neural stem cells have all been investigated in preclinical studies for their ability to generate neurons and glia [1,3–5], and the use of neural stem cells in clinical trials has been described [10].

However, there are potential dangers associated with stem cell therapy, such as malignant transformation [2,3]. The injection of pluripotent ESC or ESC-derived precursor cells in rodents leads frequently to the development of teratomas or teratocarcinomas [6,11,12], although the tumorigenic potential of ESC seems to be greatly reduced when cells are predifferentiated in vitro before implantation [7,8,13]. Importantly, ESCs seem more prone to generate tumors when implanted into the same species from which they were derived [11]. Therefore it has been noted that the absence of tumors after implantation of human stem cells into rodents does not exclude their occurrence in the human brain [2]. Recently the use of human ESC-derived dopaminergic neurons enriched by coculture with telomerase-immortalized midbrain astrocytes resulted in undifferentiated expansion suggesting there was a potential for tumor development when injected to rodents [14].

We describe here a patient affected by the neurodegenerative hereditary disorder ataxia telangiectasia (AT) who was treated by repeated transplantations of fetal neural stem cells and who developed a multifocal brain tumor.

## Methods

### Fetal Neural Stem Cell Isolation and Preparation

Isolation, preparation, and characterization of fetal neural stem cells were performed as published [15] and described in Text S1. The details are derived from a medical report describing the procedure and the protocol for the isolation and preparation of the transplanted cells, which were given to the parents of the patient.

### Pathology

The specimen was fixed in 10% neutral buffered formalin, routinely processed, and embedded in paraffin. It was cut at 5 μm for histochemical (hematoxylin–eosine, Luxol fast blue-PAS, Masson trichrome) and immunohistochemical staining (streptavidin-biotin peroxidase complex method). Antisera were directed against S-100 protein (Dako; 1:1,600, polyclonal), glial fibrillary acidic protein (GFAP) (Dako; 1:4,000, polyclonal), neurofilament protein (NF) (Dako; 1:800, 2F11), synaptophysin (ICN; 1:40, SY38), Neu-N (Chemicon; 1:10,000), epithelial membrane antigen (EMA) (Dako; 1:50, E29), p53 protein (Dako; 1:2,000, DO7), and Ki-67 antigen (Dako; 1:300, MIB-1).

### Combined Analysis of Morphology and Interphase Fluorescence In Situ Hybridization

Morphology and interphase fluorescence in situ hybridization (I-FISH) of cells obtained from touch preps of the resected tumor at the single cell level were viewed using the Duet multiparametric imaging system (Bioview) as previously described [16]. May Grunwald Giemsa (MGG)-stained touch preps were scanned, and coordinates and images of all the cells were saved for comparison with FISH analysis. Slides were destained and an I-FISH procedure with a CEP XY DNA probe kit (Vysis) was applied: X chromosome, SpectrumGreen; Y chromosome, SpectrumOrange.

The fluorescent signals in the specific cells were viewed in parallel with the morphology of the same cells scanned previously (original magnification ×1,000).

### MALDI-TOF Mass Spectrometry Genotyping of the ATM C103T Mutation and SNPs

Detection of the ATM C103T mutation [17] and genotyping of 24 SNPs including the rs7294 (VKORC1 9041G/A) [18] were carried out using the SEQUENOM MassArray MALDI-TOF mass spectrometer. Genomic DNA was extracted from blood samples, buccal smear (BS), tumor tissue, and laser capture microdissection (LCM)-isolated tumor cells. Homogenous mass EXTEND (hME) assays were designed according to the manufacturer's protocol. Each assay involves PCR amplification of the region containing the mutation/SNP of interest, Shrimp Alkaline Phosphatase treatment to remove excess dNTPs, addition of DNA polymerase along with a mixture of dideoxy and deoxy NTPs that allows extension of the hME primer through the mutation/polymorphic site and generates allele-specific extension products. This step is followed by cleanup of the extension reaction to remove salt, spotting of the extension product into 384 Sequenom proprietary SpectroCHIP, and the analysis of the spotted product using the MALDI-TOF mass spectrometry.

Sequences of the oligonucleotides used for the detection of the ATM mutation and SNPs using the Sequenom MassArray are provided in Table S1.

### PCR of Tetranucleotide Repeats and Amelogenin Homologous X and Y Alleles

Multiplex tetranucleotide repeat PCR was performed on 2 ng of genomic DNA using the AmpFlSTR SGM Plus kit (Applied Biosystems), which contains tetranucleotide repeat loci plus the X-Y homologous gene amelogenin labeled in three different colors (blue, 5-FAM; green, JOE; and yellow, NED). Amplified PCR products were subjected to capillary electrophoresis in an ABI Prism 3100 (Applied Biosystems) automated DNA sequencer using the conditions recommended by the manufacturer. The GeneScan 3.7 Analysis software (Applied Biosystems) was used for the analysis of the PCR products.

### Human Leukocyte Antigen Typing

Human leukocyte antigen (HLA) typing was determined from DNA samples extracted from peripheral blood (PB), BS, and the tumor, and from cloned amplified HLA A*, B*, and DRB1* alleles from the tumor using the primers described in the XIIth HLA Workshop proceedings and the cloning kits supplied by QIAGEN. Following the cloning procedure DNA was extracted and samples were typed for HLA A*, B*, and DRB1*. The methodologies used for HLA typing were reverse-sequence specific oligonucleotide probes (SSOP) using the Luminex platform with kits supplied by Tepnel.

### LCM of Tumor Cell Nuclei

LCM of tumor cell nuclei was performed using the PALM Microbeam laser system from 5-μm-thick sections of paraffin embedded tissue stained with hematoxylin–eosine placed on membrane-coated slides.

## Results

### Report of Case

The patient's parents gave written informed consent (as outlined in the PLoS consent form, http://journals.plos.org/plos_consent_form.pdf) to publication of his case details. In addition, the parents have confirmed to the authors that the patient is aware of the existence of the complication described in this paper and that he is aware it is discussed in a scientific publication, which is available on the internet.

A 13-y-old boy with AT, who is homozygous for the North African Jewish C103T ATM gene mutation presented to the Sheba Medical Center in February 2005 with recurrent headaches. On examination he had severe neurological deficits characteristic of AT, affecting mainly his motor functions and making him wheelchair bound. The patient's intelligence was normal and he is highly motivated at school and socially active. His recent detailed immunologic and hematological status is given in Table S2. Since the age of 7 y he has been treated for hypogammaglobulinemia at the Sheba Medical Center with monthly intravenous immunoglobulin.

In May 2001 at the age of 9 y, in March 2002 at the age of 10 y, and in July 2004 at the age of 12 y, he was taken by his parents to be treated in Moscow with repeated transplantation of fetal stem cells (see Text S1 for details as supplied to the parents by the patient's physicians in Moscow). The treating team at the Sheba Medical Center was not involved in this treatment.

**Figure 1:**
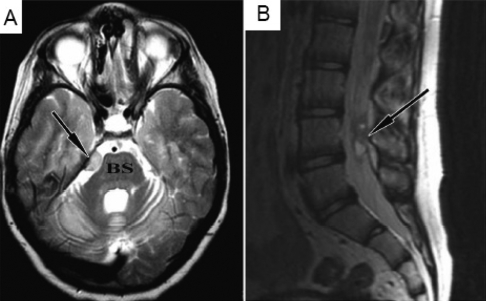
Pre-Operative MRI (A) Brain MRI (T2) demonstrating a lesion (arrow) based on the tentorium, next to the brain stem (BS). (B) Spinal-lumbar MRI (T2) showing intradural lesion (arrow), at the level of the L4 vertebra.

**Figure 2:**
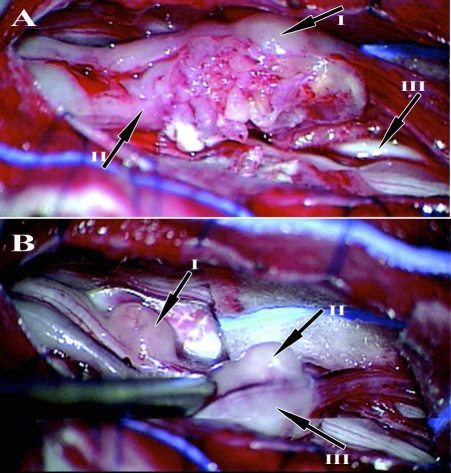
Intra-Operative View (A) The main lesion situated within the cauda equina nerve roots (I, the main tumor bulk; II, nerve root entering the tumor; III, normal nerve root). (B) One lumbar level above the main lesion, several “satellite” lesions are seen adherent to the caudal nerve roots (I, II, III, satellite tumors rostral to the main tumor bulk).

MRI performed in February 2005 to investigate the headaches revealed a right infratentorial lesion slightly compressing the brain stem and another lesion at the cauda equina (Figure 1A and 1B). The lesions grew slowly as evidenced by repeat MRIs in June and July 2006. In September 2006 at the age of 14 y, surgery was performed and a tumor localized at L3–4 level attached to the cauda equina nerve roots was removed. Additional “satellite” lesions were identified attached to nerve roots rostral to the main lesions (Figure 2A and 2B).

Since surgery the patient's general condition has remained stable. During follow up there has been slow growth of the tumor adjacent to the right infratentorial lesion with some compression on the brain stem. MRI of the infratentorial tumor at diagnosis and in January 2008 illustrates that the mass has more than doubled in volume (Figure S1). MRI of the spinal cord in July 2007 did not reveal significant growth compared to the postoperative imaging and there is no overt clinical deterioration. Based on the pathology and since patients with AT are extremely sensitive to chemotherapy and radiotherapy it was decided to follow up the patient conservatively and to repeat surgery only if indicated by clinical deterioration.

### Histology

**Figure 3:**
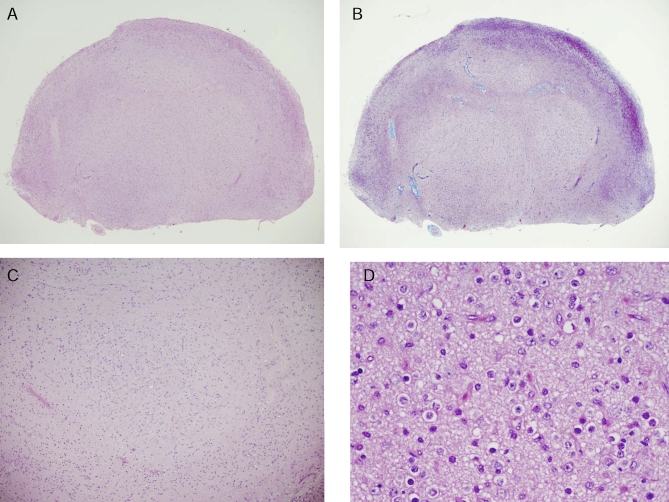
Hematoxylin–Eosine Staining of the Tumor (A) The bulk of the specimen consisted of ill-defined lobules of glioneuronal cells. (B) These cells are best seen on Masson trichome stain. (C) At medium power glioneuronal cell circumscription by astroglial cells is apparent. (D) The centers of the lobules consist of two cell types, including pale neurons with large round nuclei, open chromatin, and a distinct nucleolus as well as smaller, astrocytic cells with more hyperchromatic nuclei and pale cytoplasm.

**Figure 4:**
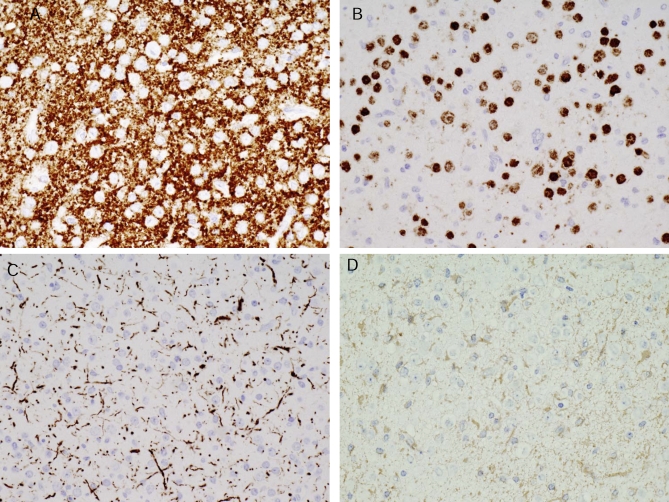
Immunohistochemistry of the Tumor (A) Immunohistochemical staining of the cells comprising the nodules show abundance of neurons as evidenced on synaptophysin, (B) neu N, and (C) neurofilament protein preparations. (D) The stromal astrocytes show cytoplasmic reactivity for glial fibrillary acidic protein.

**Figure 5:**
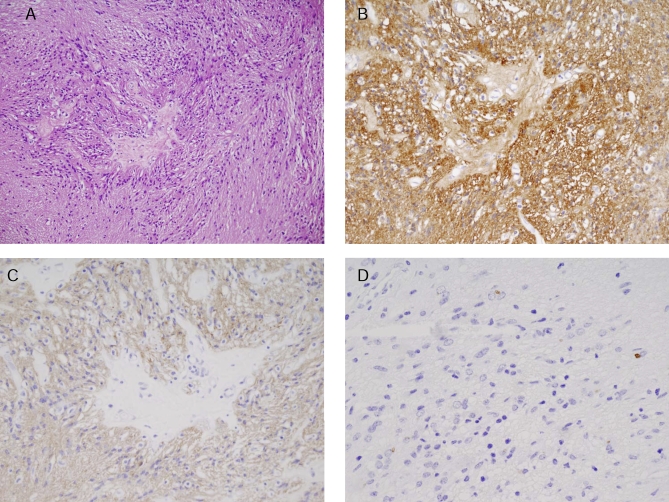
Histology and Immunohistochemistry of the Tumor (A) The minor element of the tumor consists of markedly elongate ependymal cells (tanycytes) with refractile, fibril-rich processes terminating upon the stroma. (B) Note strong staining for S-100 protein, and (C) glial fibrillary acidic protein. (D) Scattered dot-like paranuclear microlumens, a feature of ependymal cells, show epithelial membrane antigen reactivity.

The specimen consisted of three fragments of tumor ranging in size from 0.4 to 1 cm in greatest dimension. The pathology specimen (Figures 3–5) consisted almost entirely of glioneural tumor and only in small part of a remnant of the proximal filum terminale accompanied by scant benign adipose tissue, a not uncommon incidental finding at that site. No nerve root tissue was included. Much of the lesion was characterized by vague nodules of neurons and accompanying glial cells. The latter, consisting largely of astrocytes, formed a circumferential rind about the lesion and served to demarcate ill-defined aggregates of intermingled neurons and astrocytes. The neurons, all relatively mature, varied in cytologic characteristics to include (a) medium size neurons with open chromatin, small nucleoli, and relatively small quantities of pale cytoplasm lacking Nissl substance and (b) sizeable neurons with larger nuclei, distinct nucleoli, more amphophilic cytoplasm, and discernible Nissl substance. Neuronal dysmorphism, including binucleation, was infrequent. Staining for both synaptophysin and neurofilament protein was noted only in association with the neurons and their processes. Also conspicuous were two glial elements, one ependymal and the other astrocytic in nature. Comprising much on one tissue fragment, the monomorphous ependymal cells were tanycytic in appearance, featuring elongate nuclei and very long, aligned, heavily fibrillated processes. In contrast, the astrocytes possessed oval nuclei, eccentric cytoplasm, and scant processes. Immunohistochemical stains showed strong s-100 protein and glial fibrillary acidic protein (GFAP) reactivity in the glial element. Epithelial membrane antigen (EMA) staining was scant and took the form of rare, paranuclear dots within the cytoplasm of the ependymal cells.

The entire lesion was unassociated with cytologic malignancy, mitotic activity, microvascular proliferation, or necrosis. Low to moderate strength p53 staining was seen in 40% of astrocytic and 40% of ependymal cells. The MIB-1 preparation showed very low level staining (1%). Also indicative of slow lesion growth was the finding of vascular hyalinization, a feature accentuated on Masson trichome stain.

### Cytogenetic and Molecular Analysis of Tumor

**Figure 6:**
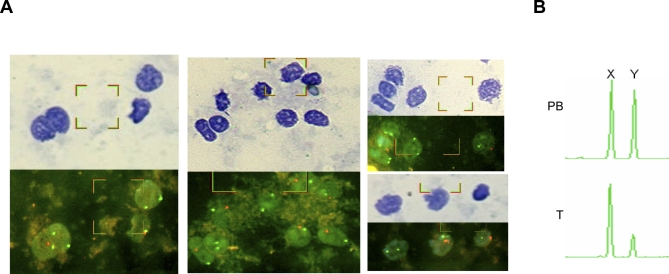
Analysis of X and Y Chromosomes of the Tumor Cells Using I-FISH and PCR Analysis of the Amelogenin X- and Y-Specific Alleles Showing the Presence of Female Cells in the Tumor (A) Combined analysis of morphology and I-FISH with X chromosome (SpectrumGreen) and Y chromosome (SpectrumOrange) probes on tumor touch preps. Upper panels, May Grunwald Giemsa-stained cells; lower panels, I-FISH analysis of the cells appearing in the upper panel. Two green signals indicate female cells (XX). One green and one orange signal indicate male cells (XY). (B) PCR analysis of the X chromosome and Y chromosome amelogenin homologous alleles in the patient's PB and tumor (T).

**Figure 7:**
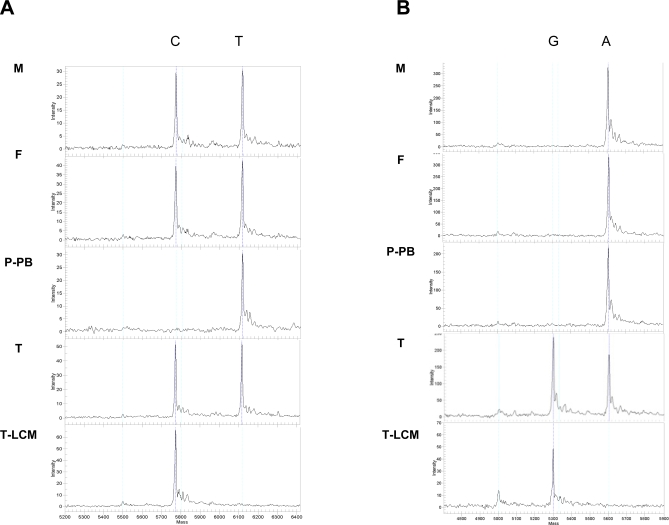
Analysis of the ATM C103T Mutation and the VKORC1 G9041A (rs7294) Polymorphism Showing Foreign Origin of the Tumor Cells (A) MALDI-TOF mass spectrometry of nucleotide 103 of the *ATM* gene using the SEQUENOM MassArray. C, wild type allele; T, the North African Jewish ATM mutated allele. F, father's PB; M, mother's PB; P-PB, patient's PB; T, tumor; T-LCM, isolated tumor cells obtained by LCM. (B) MALDI-TOF mass spectrometry of VKORC1 9041G/A (rs7294) polymorphism using the SEQUENOM MassArray. G/A, SNP at position 9041 (rs7294) of the VKORC1 gene. F, father's PB; M, mother's PB; P-PB, patient's PB; T, undissected tumor; T-LCM, isolated tumor cells obtained by LCM.

The occurrence of a tumor following administration of neural stem cells and the unusual presentation of an extra-axial multifocal glioneural neoplasm in the proximity of the injection sites raised the possibility of a donor-derived tumor. In order to explore this possibility cytogenetic and molecular approaches were used. Since the patient is affected by AT, known to be characterized by genetic instability [19], we used several complementary methodologies to prove that the nonidentity of the tumor and the patient is not due to chromosomal and genetic instability. FISH using X- and Y-chromosome specific probes on touch preps from the tumor biopsy revealed that the tumor removed from the male patient is composed of both XX and XY cells (Figure 6A). Analysis of the homologous amelogenin gene X- and Y-specific alleles in the patient's PB and tumor reveals that, while in the PB the signals from the two alleles are equal, in the tumor the X allele signal intensity is three times that of the Y allele (Figure 6B). This result further supports the presence of cells originating from a female donor. Analysis of the ATM gene North African Jewish C103T mutation using the SEQUENOM MassArray (Figure 7A) identified both parents of this patient as heterozygous for this mutation while homozygosity was demonstrated in the patient's PB and BS. Analysis of DNA extracted from undissected tumor revealed the presence of both the mutant and the normal allele. DNA extracted from LCM isolated tumor cells (Figure S2) carried only the normal allele, and the mutant allele is not detected, proving that the tumor is of donor origin. We analyzed 24 known SNPs using the SEQUENOM MassArray on DNA samples from the tumor, the patient's PB and BS, and the parents' PB (list of all SNPs tested is given in Table S1). Three SNPs, rs1043550, rs7294, and rs1061170, were informative and identified a tumor-specific allele that is not present in the patient PB or BS. One of them, rs7294 (VKORC1 9041G/A), proves conclusively that the tumor genotype could not originate from the patient or his parents. Figure 7B shows the analysis of this SNP in the DNA obtained from the tumor biopsy and in LCM isolated tumor cells, in comparison to the pattern observed in PB of the patient and his parents. The patient and his parents are homozygous for the A allele, the undissected tumor revealed the presence of both the A and G alleles, while DNA extracted from LCM isolated tumor cells carried only the G allele. This result further supports the fact that the tumor is of nonhost origin and precludes the rare possibility of tetragametic chimerism by showing that the nonhost allele is not represented in the parental germline [20].

**Figure 8:**
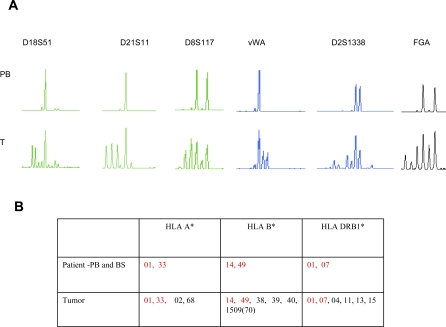
Analysis of DNA Tetranucleotide Repeats by Fluorescent PCR and Indicating the Origin of Tumor Cells from at Least Two Donors (A) Polymorphic pattern of the DNA tetranucleotide repeats of the D18S51, D21S11, D8S117, vWA, D2S1338, and FGA loci as detected by fluorescent PCR. PB, patient PB; T, tumor. (B) HLA-A*, B*, and DRB1* typing obtained by reverse-sequence specific oligonucleotide probes (SSOP) using the Luminex platform. The patient's germline HLA (determined in PB and BS) are highlighted in red. Nonself alleles are highlighted in black.

Analysis with polymorphic microsatellite tetranucleotide repeat markers of DNA from the tumor and DNA extracted from PB and BS obtained from the patient clearly showed that a number of alleles of nonhost origin are present in the tumor (Figure 8A and Table S3). The number of nonrecipient alleles detected in the tumor indicates that more than one donor cell population is present. A possible alternative explanation for the presence of additional variants in the DNA microsatellite profile in tumors is genomic instability resulting from mismatch repair defect [21]. We therefore investigated further the number of possible donors present in the tumor by detailed HLA analysis. HLA typing of the patient's PB and BS, and the tumor biopsy is shown in Figure 8B. The analysis conclusively identified the presence of two HLA A*, four HLA B*, and four HLA DRB1* nonhost alleles in the tumor. The presence of additional HLA A*, B*, and DRB1* alleles not present in the patient germline HLA or in his parents, confirms the presence of nonhost donor cells in the tumor. The presence of four additional alleles of HLA B* and DRB1* confirms the presence of cells originating from at least two donors.

## Discussion

We describe here a patient with AT who was treated repeatedly with fetal neural stem cells and who developed a multifocal brain tumor. Histology and immune phenotyping of the resected mass led to the diagnosis of a glioneuronal tumor. This is the first report of a human brain tumor developing from transplanted neural stem cells. The results obtained from the various cytogenetic and molecular studies performed on the tumor led us to conclude that the tumor is derived from the neural stem cells obtained from at least two aborted fetuses that were administered a few years earlier. This case therefore confirms that the concerns raised regarding the risk of tumor development resulting from therapeutic transplantation of neural stem/precursor cells were not unfounded.

The cellular composition of the glioneural tumor encountered in this unique case is not surprising given the use of neuronal stem cells. Glia and neurons, the two principal cell types comprising the central nervous system, were both represented and well differentiated. Unlike conventional glioneuronal tumors occurring spontaneously in the human nervous system, the glial element of this patient's tumor was bitypic, including not only astrocytes but ependymal cells as well. Ependymal cells are only rarely encountered in association with neurons in human mixed glial and neuronal tumors [22]. The benign, morphologically mature nature of the sampled tumor could only be a reflection of differentiation and maturation.

The slow but demonstrated growth of both this patient's lesions is in keeping with the low MIB-1 labeling index noted. Lacking a biopsy of the subtentorial tumor, the histogenetic relationship to the biopsied tumor remains to be determined. The similar growth rate of both processes suggests that both may be glioneuronal in nature. As separate stem cell injections were involved in the proximity of the two lesions there is no need to postulate spinal seeding of the intracranial tumor to the lumbar sac. The multiple lumbar sac deposits may be entirely independent and morphologically distinct, their occurrence representing multifocal implantation at the time of stem cell injection rather than a manifestation of seeding of a single implant*.*

The slow growth of the tumors, the benign character of the biopsied tumor, and the low MIB-1 labeling index suggest a favorable prognosis, although it should be noted that all available information regarding the clinical behavior of such tumors is based on endogenous tumors and does not necessarily reflect the clinical behavior of a tumor of exogenous origin.

While most naturally occurring tumors are monoclonal, cancers developing in exceptional situations favoring tumorigenesis are often oligo- or multiclonal. For example, embryonal tumors such as retinoblastoma occurring in individuals carrying a germline mutation in a tumor suppressor gene [23] and virally induced malignancies such as Epstein-Barr virus (EBV)-related post-transplant lymphoproliferative diseases [24] often contain several distinct clones. The therapeutic use of fetal neural stem cells derived from several donors and treated in vitro by growth factors in the described case may have created a high risk situation where abnormal growth of more than one cell occurred.

Germinal regions such as the subventricular zone have long been proposed as the areas where gliomas originate. In animal models, regions of the brain with stem cell populations are more sensitive to chemical or viral oncogenesis than areas with a low proportion of proliferating cells [25,26]. While neoplastic transformation of fully differentiated glia is widely assumed to be involved in gliomagenesis, this report describing the development of a glioneural neoplasm from transplanted neural stem cells supports an alternative mechanism, namely the development of the tumor from neural stem/progenitor cells [25,26]. It is possible that the in vitro expansion of fetal neural stem cells in conditioned medium supplemented with growth factors such as bFGF and EGF promoted transformation [27]. In addition it is possible that neuronal stem cells injected to ectopic sites where they are free of normal developmental control mechanisms provided by the physiological microenvironment may undergo abnormal growth and differentiation.

In recent years the possibility of transmission and engraftment of malignant and premalignant cells has emerged as an important mechanism for cancer development and propagation [28]. We have shown that all canine transmissible venereal tumors (TVT) originate from the same tumor and spread by malignant cell transplantation [29,30]. Another illustrative model for transplanted tumors is the Tasmanian devil facial-tumor disease [31]. Donor origin tumors have also been described in humans, in cases of maternal to fetal transmission of cancer [28,32] and in solid organ transplant recipients [33]. In the majority of such cases of tumor transplantation immune surveillance failure can be attributed to immunodeficiency. The patient described, like other AT patients, suffers from immune deficiency, which may have prevented rejection of the transplanted cells. This immunodeficiency may explain the persistence of donor cells from at least two donors several years after the experimental therapy.

In some transplanted tumor models such as the TVT, donor-derived tumor can subsequently regress because of immune rejection. Such results suggest that modulation of the immune system could be a possible future therapy in the patient described here. However, the immune deficiency of our patient is not severe (see Table S2), which suggests that even relatively immunocompetent patients may also fail to reject neural stem cells and be at risk for tumor development. In addition, a child's developing central nervous system may provide an environment that promotes tumorigenesis, especially in combination with the abnormal AT host environment characterized by deranged cellular responses to stress. Cases of astrocytoma have been reported in children with AT [34].

Studies of leukemia in monozygotic twins and screening of neonatal blood samples for leukemia-specific genetic aberrations have shown that in most childhood leukemias premalignant cells are already present in utero [35]. It usually takes months or years for frank leukemia to develop, suggesting a need for additional hit(s) for clinically evident disease to occur. It is estimated that one child out of 20 is born with a preleukemic clone [35]. Initiation of disease in utero by chromosomal translocations exceeds the rate of overt leukemia by some 100-fold. A similar ratio has been shown in putative premalignant lesions detected at autopsy by histopathology, resembling neuroblastoma and Wilms tumor compared with the actual frequency of clinically evident tumors [35]. These findings overall suggest that in several common pediatric cancers the occurrence of premalignant stem/progenitor cells might be very common prenatally, but the transition to overt disease is rare. This hypothesis may be relevant to premalignant fetal neural stem cells as well and should be borne in mind when fetal neural stem cell therapy is considered.

The only type of malignancy that has been clearly shown to develop as a result of stem cell therapy in humans is donor type leukemia following hematopoietic stem cell transplantation [36]. It appears that donor type leukemia may occur substantially more frequently with a cord blood source of stem cells than with adult bone marrow or PB stem cells [36]. Umbilical cord stem cells are more immature than their adult counterparts. Similarly, one can suggest that the less differentiated fetal neural stem cells are potentially more prone to transformation than adult neural stem cells.

Stem cell therapy and neural stem cells, in particular, provide hope to patients affected by devastating diseases. However, such innovative treatments also carry substantial risks and the potential for malignant transformation of transplanted cells has been raised [2,3]. The occurrence of a donor origin brain tumor in the patient described here provides the first proof that the concerns raised are valid. Any innovative therapeutic approach carries predictable and unpredictable risks. For example the impressive success of gene therapy for children suffering from SCID-X1 was marred by the occurrence of leukemia resulting from viral integration leading to oncogene activation [37]. Yet also “conventional” therapies such as chemotherapy, radiotherapy, and bone marrow transplantation used to treat life threatening diseases, are associated with morbidity and mortality. Our findings therefore do not imply that the research in stem cell therapeutics should be abandoned. They do, however, suggest that extensive research into the biology of stem cells and in-depth preclinical studies, especially of safety, should be pursued in order to maximize the potential benefits of regenerative medicine while minimizing the risks.

## Supporting Information

Figure S1 Head Coronal Cuts with Gadolinium Injection Showing the Tentorial Lesion on the Right Side From (A) 2005 to (B) January 2008, the tumor has grown significantly, more than doubling in volume.
(196 KB DOC)

Figure S2 LCM of Tumor Cell Nuclei (A) High power photomicrograph showing tumor cells intermingled with small capillaries (hematoxylin–eosine stain, magnification ×400). Arrows indicate the tumor cells selected for isolation by LCM.
(B) The same section following microdissection of tumor nuclei (hematoxylin–eosine stain, magnification ×400). Arrows indicate sites of removed tumor cell nuclei.
(264 KB DOC)

Table S1 Oligonucleotides Used for the Detection of the ATM Mutation and SNPs Using the SEQUENOM MassArray (46 KB DOC)

Table S2 Patient's Recent Immunological and Hematological Laboratory Data (53 KB DOC)

Table S3 Microsatellite Loci Analysis of Patient PB and Tumor Cells (27 KB DOC)

Text S1 Protocol for Stem Cell Preparation (26 KB DOC)

## Abbreviations

AT: ataxia telangiectasia
BS: buccal smear
ESC: embryonic stem cell
HLA: human leukocyte antigen
I-FISH: interphase fluorescence in situ hybridization
LCM: laser capture microdissection
PB: peripheral blood
