# Supplementary material for: Donor-Derived Brain Tumor Following Neural Stem Cell Transplantation in an Ataxia Telangiectasia Patient
Source: PLoS Med. 2009 Feb 17;6(2):e1000029. doi: 10.1371/journal.pmed.1000029 (PMC2642879; doi:10.1371/journal.pmed.1000029)
Supplement: Supplementary file 1 [file pmed.1000029.sd001.doc]

**Methods S1.**

**Stem cell preparation protocol**

According to the protocol provided to the parents by the Russian team periventricular tissue isolated from fresh-autopsy fetal brain was immediately placed on ice (+4°C) for further manipulation. After the addition of medium F12 without any supplements the crude tissue was dispersed and mechanically dissolved by manifold repeated pipetting by 5ml pipette. The resting tissue pellet resistant to disaggregation was decanted. In case of short-term storage the tissue was placed in special medium - "Hibernate E" (cat.№ 10741-023,"GibcoBRL") for 3-5 hrs preservation at +5°C. The resulted cell suspension was centrifuged at 600 rev/min (g) with the subsequent 2-fold washing of cell suspension in the medium F-12. The mean cell viability was calculated by Trypan blue and Propidium iodide tests independently. For further experiments cell specimens were selected with the level of cell viability more than 50-60%. Isolated cells were cultured in NPBM medium ("Clonetics", Cat # CC-4241, containing bFGF, EGF, NSF, gentamycin, amphotericin B). Cells were plated at cell density around 2 mln/ml. The renewal of 50% of NPBM medium was performed each 3^rd^ or 4^th^ day of culturing. Cell suspension in culture was repeatedly triturated to dissolve cell aggregates and spheres. The first lucid spherical clones were found to appear on the 3^rd^-5^th^ days of cell maintaining in NPBM medium. The number and the size of clones increased with the time of cultivation. During the first week of cell cultivation more than 40-50% of plated cells did not survive. Meanwhile the cell heterogeneity (cells of various size and form) among the survived population of cells remained steady among single cells and clones. After 2 passages (second week of culturing) the total number of spheres and the total number of single cell population increased with the time of cultivation. The culture was enriched by a new population of large-sized cells with a big nucleus released from the spheres. These subpopulations of cells were found to regularly exclude Trypan blue. The cell population was used after 3-4 passages with the total length of culturing within 12-16 days.

The neural stem cells used were derived from fetuses aborted at week 8-12. 50-100X10^6^ cells, obtained from 1-2 fetuses were given in each treatment in 2-3 cc, either by direct injection into the cerebellar white matter by open neurosurgical procedure or by injection into the patient's CSF by lumbar puncture. Infectious diseases were ruled out in the mothers and the fetuses and only karyotypically normal fetuses were used for isolation and preparation of fetal neural stem cells.
