# Supplementary material for: Donor-Derived Brain Tumor Following Neural Stem Cell Transplantation in an Ataxia Telangiectasia Patient
Source: PLoS Med. 2009 Feb 17;6(2):e1000029. doi: 10.1371/journal.pmed.1000029 (PMC2642879; doi:10.1371/journal.pmed.1000029)
Supplement: Supplementary file 2 [file pmed.1000029.sg001.doc]

**Figure S1**

**Legend to fig. S1**

Head coronal cuts with Gadolinium injection showing the tentorial lesion on the right side. From 2005 (A) to January 2008 (B), the tumor has grown significantly, more than doubling in volume.
