# Supplementary material for: Donor-Derived Brain Tumor Following Neural Stem Cell Transplantation in an Ataxia Telangiectasia Patient
Source: PLoS Med. 2009 Feb 17;6(2):e1000029. doi: 10.1371/journal.pmed.1000029 (PMC2642879; doi:10.1371/journal.pmed.1000029)
Supplement: Supplementary file 3 [file pmed.1000029.sg002.doc]

**Figure S2**

**A**

**B**

**Legend to fig. S2**

Laser capture microdissection of tumor cell nuclei

(A) High power photomicrograph showing tumor cells intermingled with small capillaries (Haematoxylin and Eosine stain, magnification x400). Arrows indicate the tumor cells selected for isolation by Laser Capture Microdissection (LCM).

(B) The same section following microdissection of tumor nuclei. (Haematoxylin and Eosine stain, magnification x400). Arrows indicate sites of removed tumor cell nuclei.
