# Supplementary material for: Donor-Derived Brain Tumor Following Neural Stem Cell Transplantation in an Ataxia Telangiectasia Patient
Source: PLoS Med. 2009 Feb 17;6(2):e1000029. doi: 10.1371/journal.pmed.1000029 (PMC2642879; doi:10.1371/journal.pmed.1000029)
Supplement: Supplementary file 4 [file pmed.1000029.st001.doc]

**Table S1**

**Oligonucleotides used for the detection of the ATM mutation and SNPs using the SEQUENOM™ MassArray -**

| RS/Position | Forward- PCR Primer | Reverse-PCR Primer | Extension Primer | TERM |
| --- | --- | --- | --- | --- |
| rs1043550 | ACGTTGGATGTACTTTTGAGGAAATGAGGG | ACGTTGGATGCAGCCAGGCCACAGATTTTG | AAATGAGGGTTCCTCCG | ACG |
| MC1R_EX1_N1619_T/C | ACGTTGGATGAAGGCCAGGCAGCAGATGAA | ACGTTGGATGACCATCGCCAAGAACCGGAA | GGCAGCAGATGAAGCAGTA | ACG |
| rs1799853 | ACGTTGGATGATGACGCTGCGGAATTTTGG | ACGTTGGATGTCAACTCCTCCACAAGGCAG | GGGAAGAGGAGCATTGAGGAC | ACG |
| rs1051740 | ACGTTGGATGTTGACTGGAAGAAGCAGGTG | ACGTTGGATGTGGCGTTTTGCAAACATACC | GCAGGTGGAGATTCTCAACAGA | ACG |
| rs17708472 | ACGTTGGATGCCCAGTCTCTGATGCAAAAC | ACGTTGGATGCCCGGCCCTTAAGTAATTC | CAAAACCGAGTGAACCGTTATAC | ACG |
| rs7294 | ACGTTGGATGTTCTAGATTACCCCCTCCTC | ACGTTGGATGAAAAAAGAGCGAGCGTGTGG | CTCCTCCTGCCATACCC | ACT |
| rs1045485 | ACGTTGGATGCGACCTTTGAAGAGCTTCAT | ACGTTGGATGTGTGGTCCATGAGTTGGTAG | TTGAGATCAAGCCCCAC | ACT |
| rs9332739 | ACGTTGGATGCCCGTACCTTTATAGTTGGC | ACGTTGGATGTGTCTGTCCTGAACGACAAC | TTTTCCAGGCTGCTGATCAC | ACT |
| CALU_EX3_N75_T/G | ACGTTGGATGCACTGTGGATGAGCTCAAAG | ACGTTGGATGTTCCACTGTCGCTCTACATC | AGCTCAAAGACTGGATTAAAT | ACT |
| rs9934438 | ACGTTGGATGACCTGGGCTATCCTCTGTTC | ACGTTGGATGTGACATGGAATCCTGACGTG | CGACCTCCCATCCTAGTCCAAG | ACT |
| rs2290228 | ACGTTGGATGCTGCCTCCTGAATTAACTGC | ACGTTGGATGACAGGGACAGGCACATAAGA | AGATCTAATTATCATGGACCTGC | ACT |
| rs4151667 | ACGTTGGATGTTCTCTCCTGCCTTCCAACG | ACGTTGGATGCAAGAGGCCCAAGATAAAGG | CAATCTCAGCCCCCAAC | CGT |
| rs547154 | ACGTTGGATGAAGTGAGGGGCACTGTGTC | ACGTTGGATGTGCTGGGTGAGTGAGCTTTG | CTGTGTCCAGGTTCCCAA | CGT |
| rs10490924 | ACGTTGGATGGAGAGAAGAAGGCTGGTAAG | ACGTTGGATGAAGCAGAGGAGCAAACTGTC | GCTCAGTGTGGATTTTAG | CGT |
| VKOC1_EX1_N129_C/A | ACGTTGGATGAAGACGCGCGAACAGCTGAT | ACGTTGGATGTCTACGCGCTGCACGTGAA | CAGAGCGCGCGGTAATCCCGGT | CGT |
| rs1057910 | ACGTTGGATGCTACACAGATGCTGTGGTGC | ACGTTGGATGTGTCACAGGTCACTGCATGG | GTGGTGCACGAGGTCCAGAGATAC | CGT |
| rs34474212 | ACGTTGGATGACTCACCCATGTACTGCTTC | ACGTTGGATGCGTCTCCAGCACGTTGCTC | TCTGCTGCCTGGCCTTG | ACG |
| rs641153_a | ACGTTGGATGGAGCCGCCTTTGATCTCTAC | ACGTTGGATGCTCTTCAGGTGTGACCACCA | AGCAGGATCCCTGGGGC | ACG |
| MC1R_EX1_N1826_C/T | ACGTTGGATGGCAGTGCGTAGAAGATGGAG | ACGTTGGATGTCATTGACGTGATCACCTGC | CGTAGAAGATGGAGATGTAG | ACG |
| rs1061170 | ACGTTGGATGGTTATGGTCCTTAGGAAAATG | ACGTTGGATGGGCAACGTCTATAGATTTACC | TTGGAAAATGGATATAATCAAAAT | ACG |
| rs641153_g | ACGTTGGATGCTCTTCAGGTGTGACCACCA | ACGTTGGATGGAGCCGCCTTTGATCTCTAC | TCCATGGTCTTTGGCCC | ACT |
| rs2228479 | ACGTTGGATGCCCATGTACTGCTTCATCTG | ACGTTGGATGAGGAGGATGACGGCCGTCT | TGGTGAGCGGGAGCAAC | ACT |
| rs1805005 | ACGTTGGATGATCTCTGACGGGCTCTTCCT | ACGTTGGATGTTCCGGTTCTTGGCGATGG | TGGTGGAGAACGCGCTGGTG | ACT |
| rs4986790 | ACGTTGGATGAGCATACTTAGACTACTACC | ACGTTGGATGCACACTCACCAGGGAAAATG | ACTTAGACTACTACCTCGATG | ACT |
| ATM_EX3_N31_C/T | ACGTTGGATGGAAGTTGAGAAATTTAAGCG | ACGTTGGATGTGTTTGGAATCTGAATGCCG | AAATTTAAGCGCCTGATT | ACG |

Columns, from left to right, show the SNP tested (by RefSNP number or nucleotide position from the start of the relevant exon), Forward PCR primer, Reverse PCR primer, Extension primer, ddNTP’s composition of the termination Mix.
