# Supplementary material for: Donor-Derived Brain Tumor Following Neural Stem Cell Transplantation in an Ataxia Telangiectasia Patient
Source: PLoS Med. 2009 Feb 17;6(2):e1000029. doi: 10.1371/journal.pmed.1000029 (PMC2642879; doi:10.1371/journal.pmed.1000029)
Supplement: Supplementary file 5 [file pmed.1000029.st002.doc]

**Table** **S2**

**Patient's recent Immunological and Hematological Laboratory data**

|  | Results | Reference Range |
| --- | --- | --- |
| **Complete Blood Count** |  |  |
| Hematocrit (%) | 38 | 39.8-52.2 |
| Hemoglobin (g/dl) | 12.1 | 13.5-17.5 |
| Mean corpuscular volume(μm^3^) | 76 | 80-100 |
| White cell count (/mm^3^) | 7390 | 4000-11000 |
| **Differential count** |  |  |
| Neutrophils (%) | 65 | 40-74 |
| Lymphocytes (%) | 17.4 | 20-40 |
| Monocytes (%) | 6.2 | 3-9 |
| Eosinophils (%) | 9.4 | 0-7 |
| Basophils (%) | 0.7 | 0-1.5 |
| Platelets count (/mm^3^) | 440000 | 130000-440000 |
| **Immunophenotyping** |  |  |
| WBC (/mm^3^) | 8870 | 4000-11000 |
| Lymphocytes (%) | 7.8 | 20-40 |
| Lymphocytes (/mm^3^) | 692 | 1000-4800 |
| CD3 (%) | 75 | 60-85 |
| CD3 (/mm^3^) | 519 | 960-2577 |
| CD4 (%) | 31 | 36-63 |
| CD4 (/ mm^3^) | 214 | 436-1394 |
| CD8 (%) | 33 | 15-40 |
| CD8 (/mm^3^) | 228 | 166-882 |
| CD4/CD8 | 0.94 | 0.5-3.3 |
| CD20 (%) | 3 | 5-25 |
| CD20 (/ mm^3^) | 17 | 50-300 |
| CD56 (%) | 17 | 6-30 |
| **Immunoglobulin levels*** |  |  |
| IgG (mg/dl) | 440 | 650-1600 |
| IgA (mg/dl) | 6.67 | 68-423 |
| IgM (mg/dl) | 102 | 50-300 |
| **Response to mitogens (cpm)** | **Patient (cpm)** | **Control** |
| No Mitogen | 1700 | 2100 |
| PWM (25μg/ml) | 15000 | 11000 |
| PHA (6 μg/ml) | 5200 | 70000 |
| PHA (25μg/ml) | 12000 | 88000 |
| Anti-CD3 | 52000 | 22000 |

*Measured before intravenous immunoglobulin administration
