# Supplementary material for: Donor-Derived Brain Tumor Following Neural Stem Cell Transplantation in an Ataxia Telangiectasia Patient
Source: PLoS Med. 2009 Feb 17;6(2):e1000029. doi: 10.1371/journal.pmed.1000029 (PMC2642879; doi:10.1371/journal.pmed.1000029)
Supplement: Supplementary file 6 [file pmed.1000029.st003.doc]

**Table S3**

| Locus  designation | Patient PB  alleles (bp) | Tumor  alleles (bp) |
| --- | --- | --- |
| D18S51 | 308 | 292,296,308 |
| D21S11 | 214 | 200, 204,208, 214 |
| D8S1179 | 148,156 | 140,144,148, 152,156 |
| vWA | 174 | 174,178,182 |
| D2S1338 | 320,324 | 298,302,312,316,320,324 |
| FGA | 236,244 | 224,228,232,236,240,244 |

**Legend to table S3**

Microsatellite loci analysis of patient PB and Tumor cells

Tetranucleotide microsatellite repeats PCR amplified using the AmpFlSTR SGM Plus kit (Applied Biosystems). Locus designation depicts the microsatellite analyzed. The PB and Tumour alleles are given in bp as determined using the GeneScan 3.7 Analysis Software (see legend to figure 4A).
